# Free-Colloidal Probe Lateral Force Microscopy (fCP-LFM) for Nanotribology of Sliding and Rolling Contacts

**DOI:** 10.1007/s11249-026-02147-8

**Published:** 2026-05-19

**Authors:** Simon Scherrer, Noé Margni, Kristian Skailand, Minghan Hu, Robert W. Style, Shivaprakash N. Ramakrishna, Lucio Isa

**Affiliations:** 1 Department of Materials, ETH Zürich, Leopold-Ruzicka-Weg 4, 8093 Zürich, Zürich Switzerland; 2 Department of Mechanical and Process Engineering, ETH Zürich, Tannenstrasse 3, 8092 Zürich, Zürich Switzerland

**Keywords:** Rolling friction, Sliding friction, Nanotribology, Microparticles, Microfluidics, Lubrication

## Abstract

While sliding friction at the nanoscale is routinely measured by colloidal probe lateral force microscopy (CP-LFM), measuring rolling friction at this scale remains challenging due to the difficulty of resolving lateral forces and particle rotation under well-defined normal loads. Here, we present an extension of a recently published platform that uses free-colloidal probes to enable quantitative measurements of both sliding and rolling friction at individual particle–substrate contacts. Our approach combines a custom-made colloidal probe with a holder for free particle rotation with lateral force detection and optical particle tracking. The modular holder design is compatible with particles of different sizes and materials, enabling studies of many systems of interest. We functionalize the holder surface using a lubricious poly(ethylene-glycol) (PEG) brush to minimize the internal friction of the free-moving particle within and extend the regime in which rolling friction can be measured. In addition, we present a scalable synthesis route for smooth, polymeric particles with tunable size (3–8 μm) and anisotropic fluorescence, enabling direct tracking of particle rotation without modifying native surface properties. Together, these developments establish free-colloidal probe LFM, or fCP-LFM, as a robust and adaptable tribological platform for quantifying friction at the microscale under different modes of relative motion, suitable for systematic studies of nanoscale particle–surface interactions across a wide range of materials and conditions.

**Supplementary Information:**

The online version contains supplementary material available at https://doi.org/10.1007/s11249-026-02147-8.

## Introduction

Sliding and rolling contacts govern the mechanical response of a wide range of industrial and natural systems, including foods, cementitious materials, pharmaceuticals, and consumer products [1–3]. In granular and particulate materials, macroscopic behavior often originates from microscopic particle–particle contacts, where surface forces, adhesion, and friction dictate contact evolution under load [4, 5].

While sliding friction of these contacts is well understood in the tribology and particulate matter community [6, 7], rolling friction (the resistance to rotational motion at contact) remains comparatively under-explored at the microscale. This is a notable gap, as rolling motion plays a central role in force transmission in particulate systems and contributes to phenomena such as shear thickening, shear jamming, and granulation [8–15]. A detailed understanding of the same contact mechanics at small scales is also essential for the functionality of systems such as micro-electromechanical systems (MEMS) [16] and emerging micro-robotic technologies [14, 15, 17]. An understanding of rolling friction of microparticles is therefore necessary for the engineering of colloidal systems across a wide range of applications.

The primary reason for this gap is methodological. Atomic Force Microscopy (AFM), and particularly colloidal probe lateral force microscopy (CP-LFM or CP-AFM), is the standard technique for quantifying sliding friction at the nano- and microscale [18–21]. However, in conventional implementations, the particle is rigidly fixed to the cantilever, prohibiting rotation and therefore restricting measurements to sliding motion only. As a result, rolling resistance measurements under controlled normal load have remained largely inaccessible.

Nevertheless, several experimental approaches have been proposed to probe rolling friction at the nanoscale, most of them also relying on AFM-based manipulation and force sensing. Early studies inferred rolling resistance from the deformation of particle chains compressed with AFM cantilevers [22] or optical tweezers [23–25], while subsequent work, initiated by Sitti et al. [26–28], used sharp AFM tips to push individual sub-μm particles and differentiated between rolling, sliding, or spinning based on lateral force signals and contact mechanics models [29]. Although powerful, this approach generally lacks direct detection of particle rotation and control over the applied normal force. Direct visualization has been achieved for selected systems using fluorescence microscopy [30] or SEM [31], but these methods are limited by restricted environments and low temporal resolution. Alternative strategies use rolling of particles between flat surfaces in a nano-indenter [32], or non-contact excitation [33–36]. Together, all of these rolling friction measurements remain highly system-dependent and rely largely on indirect inference of the motion state from the friction, which explains why (unlike sliding friction) no standardized, widely adopted methodology for rolling friction at the nanoscale has emerged yet.

To overcome this limitation, we recently introduced a free-colloidal probe LFM (fCP-LFM) method [37]. In this approach, we reversibly capture a particle inside a cavity within a micro-fabricated holder attached to a standard AFM cantilever, and apply a defined normal force. As we translate the cantilever laterally, the particle is pushed across the counter-surface, resulting in a frictional contact. We record normal and lateral forces via cantilever deflection and torsion, analogously to conventional CP-LFM. Since rotation is not imposed but emerges from contact mechanics, the particle may slide, roll, or exhibit mixed behavior. We quantify particle motion by combining LFM measurements with inverted fluorescence (confocal) microscopy and anisotropically labeled fluorescent particles. Rotational tracking enables a direct correlation between the local motion of the particle and the instantaneous force response, and provides insight into the origins of rolling friction.

The original implementation [38] of fCP-LFM demonstrated the feasibility of the method and introduced an analytical framework to extract rolling friction coefficients, but was restricted to a single model system of silica particles for direct comparison with previous studies on shear-thickening suspensions [6, 7]. Consequently, several practical and methodological aspects remained unaddressed, including applicability to different particle sizes and materials, as well as optimization of probe fabrication and assembly steps. As a result, robustness and throughput continued to be limited. Furthermore, open questions remained regarding the limitations in sensitivity as a result of internal friction at the particle–holder contact, which is an intrinsic component of the technique.

In the present work, we revisit fCP-LFM and present it as a general nanotribology platform, explicitly addressing the issues mentioned above. In particular, we introduce (i) a particle-synthesis route based on microfluidic-droplet generation and solvent evaporation, yielding smooth polymer particles with tunable diameters (3–8μm) and built-in fluorescence anisotropy, expanding the library of available fluorescently labeled particles for 3D rotation tracking; (ii) a modular and improved holder design fabricated by two-photon direct laser writing (2PP-DLW), enabling adaptation to different particle sizes through simple CAD modification and straightforward assembly; and (iii) a targeted surface-functionalization strategy to control internal friction at the particle–holder contact independently of the particle–substrate contact. We apply a PEG brush coating to the holder, which dramatically reduces particle–holder friction, providing reliable rolling friction values with high sensitivity to characterize low-friction, particle–substrate combinations. Together, these developments transform fCP-LFM into a versatile and sensitive tool for quantifying rolling friction at the microscale.

Moreover, this paper is intended to be a practical guide for tribologists interested in implementing the technique on their systems of interest. The specific particle–substrate system studied here serves to illustrate the capabilities of the method and to demonstrate how sliding- and rolling-dominated regimes can be experimentally accessed and distinguished.

## Methods

We present a practical guide for the implementation of the methodology, highlighting alternative options and relevant resources where applicable. In our terminology, the *probe* consists of a tip-less AFM cantilever with a micro-fabricated holder attached to its end. We use the probe to study particle–substrate interactions by reversibly capturing individual particles within the cavity of the holder. First, we explain the preparation of the probe 2.1, 2.2. Next, we provide examples for the fabrication of particles 2.6 and substrates 2.7. However, these can be tailored to the system of interest. Other sections in the methodology 2.3, 2.4, and 2.5 are not necessary to perform a rolling friction measurement but are useful to provide control data. Finally, we report the protocol for a typical rolling friction measurement 2.8, go through the analysis of particle motion 2.9 and the extraction of the rolling resistance using an analytical model 2.10.

All chemicals were used as received by the supplier, unless otherwise stated. Poly(methyl-methacrylate) (PMMA, 15000 g/mol), chloroform (99.8 %), CdSe/ZnS core–shell-type quantum dots (stabilized with octadecylamine ligands, fluorescence $\lambda _{em}$ = 520 nm, solid), sodium dodecyl sulfate (SDS, 98.5 %), 3-(trimethoxysilyl)propyl methacrylate (98 %), acetone (99.8 %), propyleneglycol monomethyl ether acetate (PGMEA, 99.5 %), 2-propanol (IPA, 99.5 %), ethanol (96 %), polyethyleneimine (PEI, ∼10000 g/mol), ammonia (25 % in water), tetraethyl orthosilicate (TEOS, 99 %), O-(2-mercaptoethyl)-O’-methylpolyethylene glycol (PEG-thiol, 2K, 6K, and 10K g/mol), and N-(2-hydroxyethyl)piperazine-N’-(2-ethanesulfonic acid) (HEPES, 1 M) were purchased from Sigma-Aldrich, Switzerland.

### Fabrication of Free-Colloidal Probes

The probe fabrication follows our previous protocol [37] with several improvements. We designed a 3D model of the holder, customized to the desired particle size, in AutoCAD 2022 and exported it as an STL file. All STL files are available at https://doi.org/10.3929/ethz-c-000792163.

We fabricated the holders using a two-photon polymerization direct laser writing system (2PP-DLW, Photonic Professional GT-2, NanoScribe, Germany), by printing them onto a fused silica substrate. As access to these types of tools is not widely available, it is also possible to contact us to provide interested users with a set of holders.

**Fig. 1:**
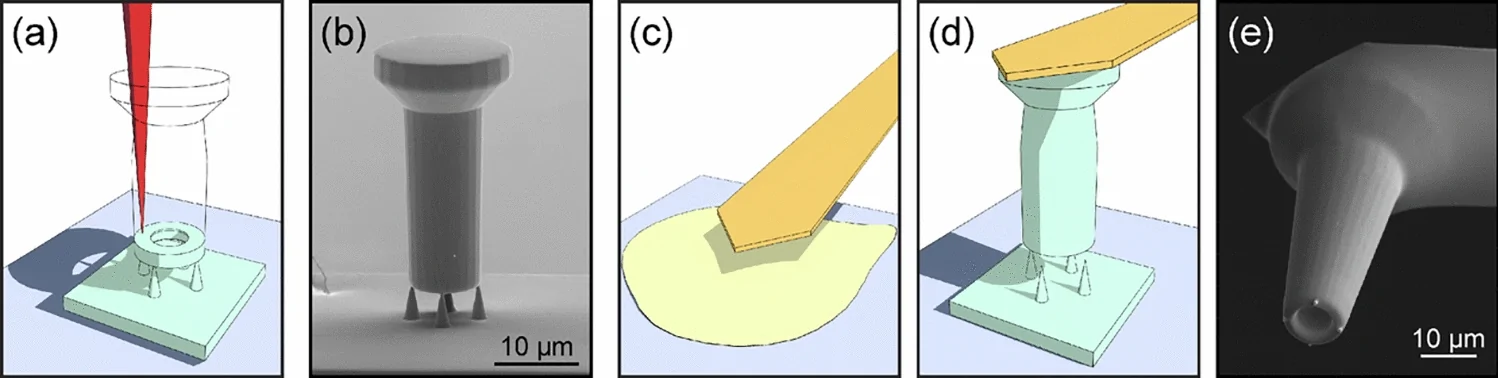
Overview of the probe assembly process. a) Schematic of the initial printing of the holder using 2PP-DLW. b) SEM micrograph of a holder (designed for 7.9 μm particles) after printing. c) Schematic depicting the pickup of UV-adhesive at the tip of the AFM cantilever. d) Schematic depicting the breaking off of the holder from the support cones after attaching it to the cantilever. e) SEM micrograph of typical free-colloidal probe after assembly (designed for 3.1 μm particles)

We rinsed the fused silica substrates (Multi-Dill, NanoScribe GmbH, Germany) with ethanol and plasma-treated them for 20 s in air (Piezobrush PZ2, Relyon Plasma GmbH, Germany). For improved adhesion, we silanized the substrates by immersion in 30 mL of acetone containing 150 μL of 3-(trimethoxysilyl)propyl methacrylate for 1.5 h, followed by ethanol rinsing and nitrogen drying. The holders were printed by 2PP-DLW with a 63 × objective of NA = 1.5 (Zeiss, Germany) and IP-S photoresin (NanoScribe GmbH, Germany). Typically, we produced batches of 15–20 holders on a single substrate using the recommended settings for IP-Dip resin (NanoScribe GmbH, Germany), adjusting only the base laser power to 50 % to account for the different resin used (Fig. 1a). We developed the prints in PGMEA (20 min), rinsed them in IPA (5 min), dried them under nitrogen, and post-cured them under 365 nm UV light for 1 h. The quality of the print was examined by optical microscopy (BX41, Olympus, Japan) and SEM (Gemini Leo-1530), as shown in Fig. 1b (displaying a holder designed for 7.9 μm particles).

We attached the printed holders to tip-less AFM cantilevers (HQ/CSC38/Cr-Au, MikroMasch, Bulgaria). We measured the normal and torsional resonance frequencies and quality factors by AFM in air and water (Nanowizard III, JPK, Germany) to calibrate the cantilevers, and found the resulting normal and torsional spring constants to be ∼0.05 N/m and $\sim 3\cdot 10^{-9}$ Nm, respectively [39, 40]. We UV-ozone cleaned (185/254 nm LED, 15 mW/cm$^2$, Ossila Ltd., UK) the cantilevers and printed holders for 10 min. A small droplet of UV-curable adhesive (Norland Optical Adhesive 81, USA) was placed on a glass slide. Using the AFM in contact mode, we brought the tip of the cantilever into contact with the droplet to transfer some of the glue to the cantilever (Fig. 1c). We then aligned the cantilever above the printed holder, brought it into contact with ∼10 nN normal force, and cured it using a UV flashlight (GEM10 UV, 3000 mW at 365 nm, Nitecore, Germany) for 1 min at a distance of 3–4 cm. We released the printed holders from the substrate by retracting the cantilever (Fig. 1d) and breaking the support cones that connect the holder to the base. The alignment and integrity of the probes were confirmed using optical microscopy and SEM, as seen in Fig. 1e (showing a probe designed for 3.1 μm particles).

### Surface Functionalization of Free-Colloidal Probes with PEG-thiol

We functionalized the assembled probes with PEG brushes to control the frictional properties at the particle–holder contact. We cleaned the holders (already mounted on cantilevers) and photoresin substrates with UV-ozone for 10 min, and then coated them with 3 nm chromium and 50 nm gold in a thermal evaporator (MED 020 coating system, BALTEC, Liechtenstein). After air-plasma activation (Zepto, Diener electronic GmbH & Co. KG, Germany) for 60 s, we immersed the samples in 4 mM ethanolic solutions of PEG-thiols (MW = 2K, 6K, or 10K g/mol) at 35 °C for 12 h [41–44]. After being rinsed and nitrogen-dried, the samples were ready for use.

### Fabrication of Fixed-Colloidal Probes

We fabricated standard fixed-colloidal probes to measure the sliding friction of PMMA particles. We deposited PMMA particles (7.9 μm) onto a clean glass slide at low concentration and dried them. We cleaned a tip-less AFM cantilever (HQ/CSC38/Cr-Au, MikroMasch, Bulgaria) with UV-ozone for 10 min and determined its spring constants as above. We deposited a small volume of UV-curable adhesive (Norland Optical Adhesive 63, USA) on the cantilever using a tungsten needle (Imina Robotics, Switzerland). We picked up a single PMMA particle with another needle and positioned it on the cantilever tip. The procedure was performed using a micropositioning system (STM-3, Lang GmbH, Germany). We cured the adhesive with UV light (GEM10 UV, 3000 mW at 365 nm, Nitecore, Germany) for 1 min and stored the probes until use.

### Fabrication of Photoresin Substrates

We fabricated flat IP-S photoresin substrates via 2PP-DLW using the same procedure as for holder fabrication. The printed structures are 100 × 100 × 10 μm slabs. We functionalized the substrates with PEG-thiol using the same procedure as for the free-colloidal probes (see 2.2). We measured the dry layer thickness of the 10K PEG brush to be ∼8 nm by ellipsometry (M-2000 Ellipsometer, J.A. Woollam Co., USA).

### Fixed-Colloid Friction Measurements

**Fig. 2:**
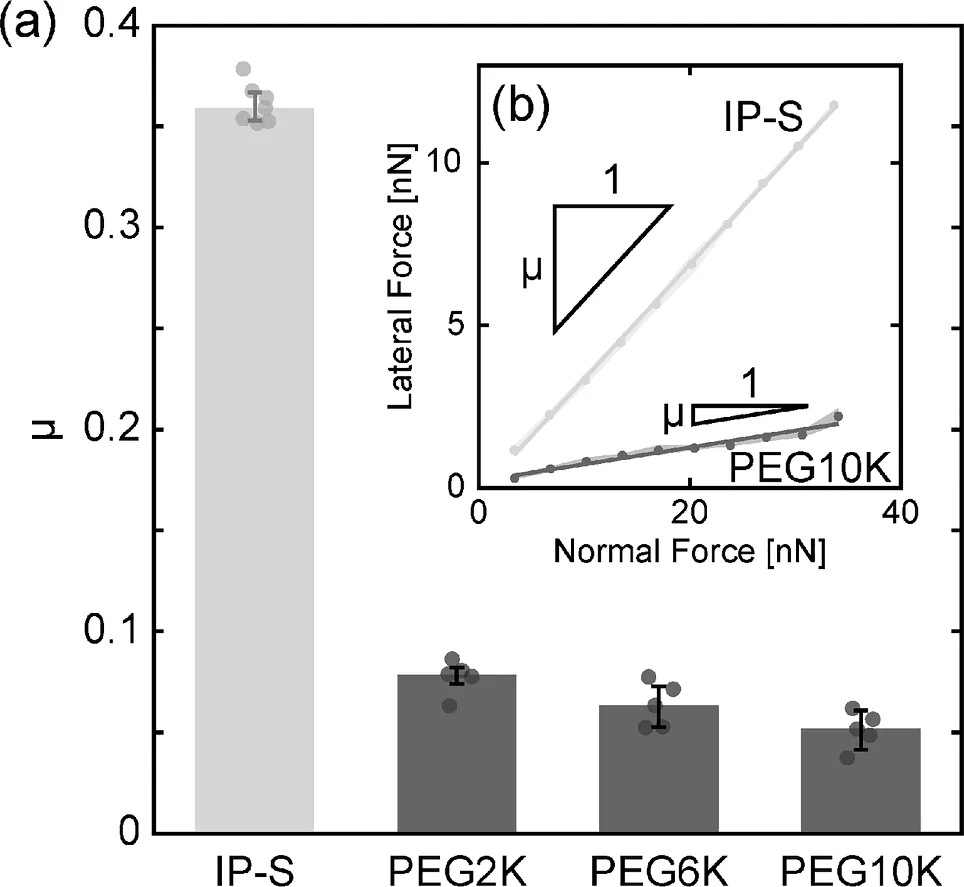
a) Sliding friction coefficients of a PMMA particle (7.9 μm) on the bare photoresin surface (IP-S) and the PEG-functionalized photoresin surface in HEPES buffer for different PEG-thiols of different molecular weights. b) The friction coefficient is determined as the slope of the linear fit to the lateral versus normal force data

We measured the sliding friction of PMMA particles against the pristine and PEG-functionalized IP-S photoresin surface in HEPES buffer (10 mM) on an AFM (MFP-3D, Asylum Research, Oxford Instruments, UK), as seen in Fig. 2. We cleaned the colloidal probe with UV-ozone for 10 min and submerged the substrate in buffer. For each substrate, we measured friction at ≥5 locations and at 10 different normal forces at 10 μm/s.

### Fabrication of Smooth Polymeric Particles with Controlled Size and Anisotropic Fluorescence

**Fig. 3:**
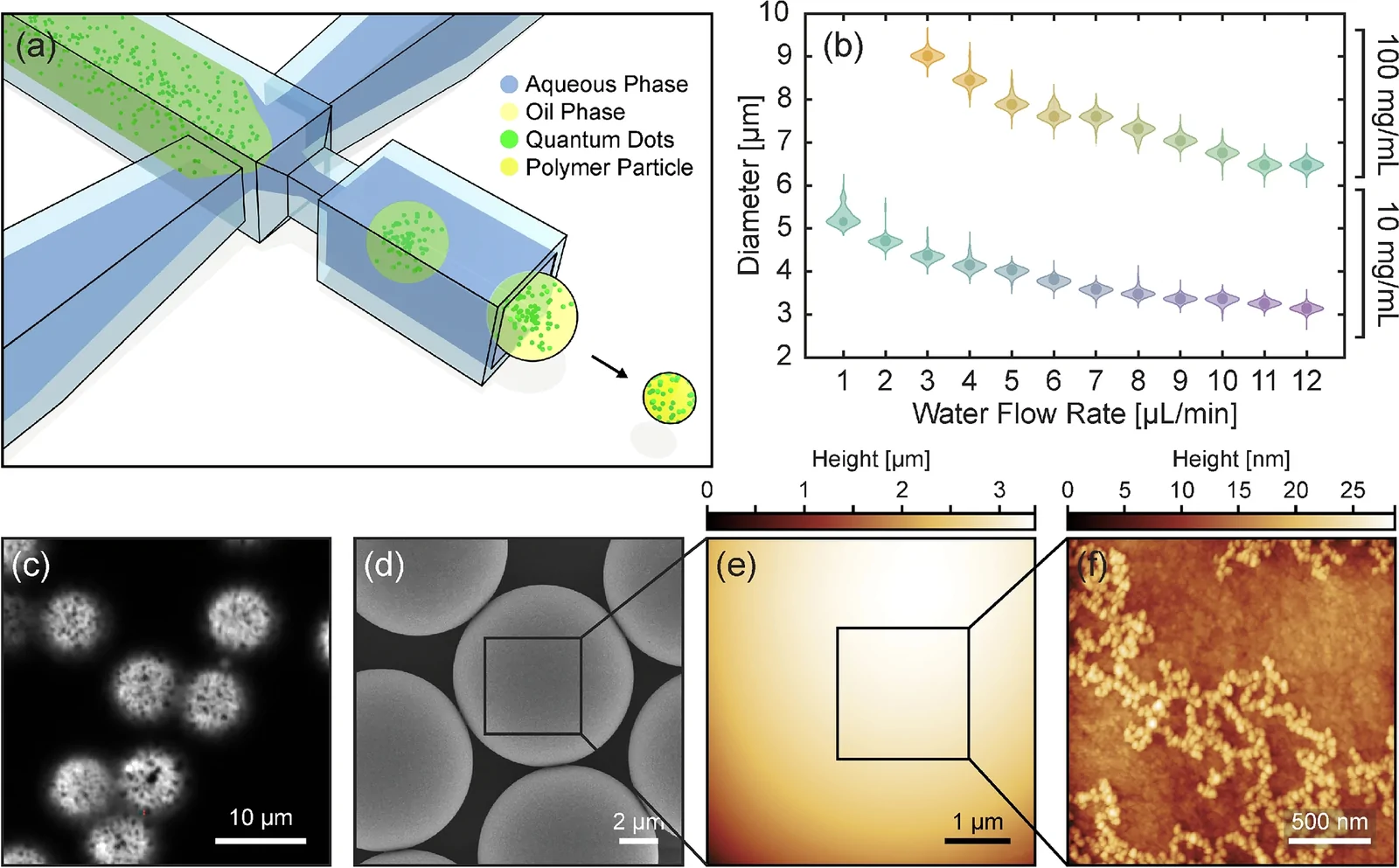
Particle fabrication via droplet formation and solvent evaporation. a) Sketch, depicting the droplet formation within a microfluidic chip using a flow-focusing geometry. b) Final particle diameters for two initial polymer concentrations (100 and 10 mg/mL), as a function of the water flow rate, extracted from SEM scans. Violin plots indicate the corresponding size distribution for each flow-rate/concentration condition. c) Confocal microscopy scan, highlighting the inhomogeneous fluorescence on the particle surface used for rotational tracking (example of particles synthesized with 100 mg/mL PMMA, water flow rate: 3 μL/min). d) Scanning electron microscopy image of the same particle batch. e) Atomic force microscopy scan of a single particle. f) Close-up AFM scan of a surface of a single particle, with the global curvature of the particle removed to highlight the small-scale roughness caused by the quantum dots on the surface

We produced solid PMMA microparticles with inhomogeneous fluorescence via a solvent-evaporation method using monodispersed droplets generated in a flow-focusing microfluidic device [45–47]. Droplets of an oil phase containing dissolved polymer and fluorescent quantum dots were formed in an aqueous surfactant solution (Fig. 3a). As the solvent diffused into the continuous phase, the concentration of polymer inside the droplet increased until precipitation occurred, forming solid particles.

The design and fabrication of microfluidic chips follows previous work by Hu et al. [48]. The oil phase contains 100 mg of PMMA, dissolved in 950 μL of chloroform, followed by the addition of 50 μL of a quantum dot dispersion (10 mg/mL of chloroform) to achieve a final quantum dot loading of 0.5 wt%, relative to the mass of the polymer. We prepared the low-concentration polymer solution (10 mg/mL) by dilution with chloroform. The aqueous phase contains 0.2 wt% SDS, prepared by dissolving 100 mg of SDS in 50 ml of MilliQ water. We filtered both phases (0.2 μm pore size) before use, loaded them into glass syringes, and pushed the solution into the cross-flow junction using syringe pumps (Nemesys S, Cetoni, Germany) at controlled flow rates.

We monitored the formation of drops with an optical microscope (AZ100M, Nikon, Japan) as shown in Fig. S1. We fixed the oil flow rate at 1 μL/min, while varying the aqueous-phase flow rate from 1–12 μL/min to change the droplet size. We collected the droplets in an aqueous surfactant solution and gently agitated them on a rotary shaker (Unimax 1010, heidolph, Germany) for 1 h to prevent coalescence and promote solvent removal. A representative video of the particle formation process is provided in the Supplementary Information (video S1).

We repeatedly washed the resulting particles in MilliQ water to remove residual SDS. We quantified the particle size using scanning electron microscopy (SEM, Gemini Leo-1530, Germany). Using polymer concentrations of 100 and 10 mg/mL and aqueous-phase flow rates of 1–12 μL/min, we obtained mono-disperse particles 3–8μm in diameter (Fig. 3b).

Confocal microscopy (Yokogawa CSU-W1, Japan; Axio Observer D1, 63 × NA = 0.75 objective, Zeiss, Germany; Prime 95B, Teledyne Photometrics, USA) confirmed that quantum dots assemble randomly at the surface of the particle, producing a “patchy” fluorescence pattern suitable for rotational tracking (Fig. 3c). The SEM scans showed smooth spherical particles (Fig. 3d). AFM scans (Nanowizard III, JPK, Germany with OMCL-AC160TS cantilevers from Olympus, Japan, in AC mode) revealed sub-10 nm asperities attributed to surface-adsorbed quantum dots only after subtracting the global curvature of the particle shape (Fig. 3e,f).

### Fabrication of Rough Substrates

We prepared rough substrates following Hsu *et. al.* [6]. We sonicated glass cover slips (18 × 18 mm, #1, Menzel Gläser, Germany) in toluene, IPA, ethanol, and MilliQ water (10 min each), then UV-ozone cleaned them for 20 min. Next, we charged their surface positively by immersion in 1 mg/mL PEI in MilliQ water for 30 min, rinsed them with more MilliQ water and dried them under a nitrogen jet.

The substrates were then immersed in a 0.001 wt% aqueous silica nanoparticle dispersion (8 nm, NexSil 8, Nyacol, USA) for 15 min to allow for electrostatic adsorption. To permanently immobilize the particles, we transferred the substrates to a TEOS-based fixing solution containing 7.44 mL ethanol, 1.22 mL ammonia, 1 mL MilliQ water, and 0.6 mL 1 % ethanolic TEOS (freshly prepared), after a gentle wash, and stirred for 30 min. Finally, we rinsed and dried the substrate and characterized them by AFM (see Fig. S2).

### Free-Colloidal Friction Measurements

Free-colloidal friction measurements follow our previously reported method [37]. We mounted a rough substrate 2.7 on an inverted microscope (Yokogawa CSU-W1, Japan; Axio Observer D1, 63 × NA = 0.75 objective, Zeiss, Germany; Prime 95B, Teledyne Photometrics, USA) and covered it with a dilute particle suspension 2.6 in 10 mM HEPES buffer. After the particles had sedimented onto a microscope slide, we mounted the free-colloidal probe on the AFM head (Nanowizard III, JPK, Germany) and placed it on the microscope stage.

First, we identified a particle-free area of the substrate and brought the probe into contact with the surface at a low normal force (∼10 nN) to center the holder within the field of view of the optical microscope. We then retracted the probe by 30 μm. Subsequently, we centered a particle of interest in the field of view by translating the sample stage, and aligned the particle and the holder vertically by adjusting the focal plane. Finally, we captured the particle by approaching the substrate again at the desired normal force. Depending on the alignment accuracy, the particle snapped into the cavity of the holder. After capture, we performed friction measurements for each particle at six normal force setpoints over distances equivalent to two full revolutions of the particles, depending on the particle size. We adjusted the scan speeds to match the rotational velocity between particle sizes: 2.6 μm/s (3.1 μm particles), 4.4 μm/s (5.3 μm particles), and 6.6 μm/s (7.9 μm particles). At the end of each measurement, we released the particle by withdrawing the probe from the surface. In most cases, the particle dropped out of the cavity due to gravity. If detachment did not occur, we lifted the AFM head to break the liquid meniscus, reliably releasing the particle.

During scanning, we recorded confocal time series (488 nm excitation, 510–540 nm emission filter, 50 ms acquisition) to track particle rotation. We repeated the experiments using the same probes before and after surface functionalization with PEG-thiol for the three particle sizes (3.1, 5.3, 7.9 μm).

### Analysis of the 3D Rotational Motion

We analyzed the rotation of the particles following Niggel *et. al.* [49]. For each time step, the fluorescence pattern was mapped onto a sphere and virtually rotated. The best-fitting rotation relative to the subsequent time step yielded the angular displacement. The noise floor, determined from stationary particles trapped but not translated by the probe, corresponds to ∼1°  and was used as the rolling threshold. We compared the cumulative angular displacement with the theoretical rotation (i.e., corresponding to 720°for two full revolutions) to calculate the mean rotation (Fig. 5a).

### Analysis of the Lateral Force Signal

The lateral force analysis followed standard CP-LFM procedures. We converted raw voltages to forces using the calibration factors determined before the measurement and extracted average friction forces for each applied normal force. For fixed particles and free particles with <50 % mean rotation, we determine the sliding friction coefficient $\mu _{s}$ directly from the slope of the linear fit of the lateral force $F_L$ and the normal force $F_N$ following Amontons’ law [50]:

1 $$\begin{aligned} F_L = \mu _{s} \cdot F_N \end{aligned}$$

**Fig. 4:**
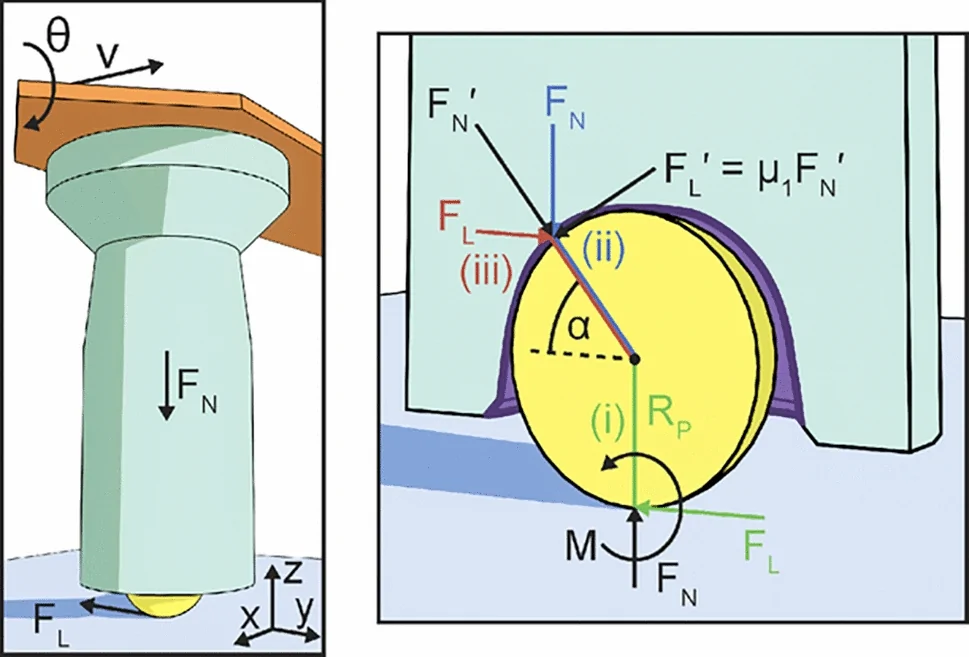
Schematic of a particle inside the holder during lateral translation. The zoom-in shows the relevant forces and moments that the holder and the substrate exert on the particle

During rolling (>50 % mean rotation), the substrate induces a torque *M* on the particle, opposing the rolling motion (Fig. 4). In analogy to Amontons’ classic sliding friction law, we can determine the dimensionless rolling friction coefficient $\eta /R_p$, given by

2 $$\begin{aligned} \frac{M}{R_p} = \frac{\eta }{R_p}\cdot F_N \end{aligned}$$

with the particle radius $R_p$. As the output of the friction measurement only yields $F_L$, we need to consider the contribution of $\mu _1$ at the particle–holder contact to determine $M/R_p$. For steady rolling, torque balance gives

3 $$\begin{aligned} M/R_p = F_L - F_N\cdot \cos \alpha + F_L \cdot \sin \alpha \end{aligned}$$

where α is the polar angle at the particle–holder contact point, relative to the substrate plane and depends on the applied normal force $F_N$ (see Fig. 4). The last two terms on the right side of the Eq. 3 come from the torque generated at the particle–holder contact, and are associated with the particle–holder sliding friction coefficient $\mu _1$, also following Amontons’ law:

4 $$\begin{aligned} F_L' = \mu _1\cdot F_N' \end{aligned}$$

With $F_L'$ and $F_N'$ being the lateral force and normal force acting at the particle–holder contact, respectively. Both $F_L'$ and $F_N'$ are determined by $F_L$ and $F_N$, based on α (see Fig. 4). Having measured $\mu _1$ independently (see Fig. 2) and substituting $F_L'$ and $F_N'$ in Eq. 4, we can use it to calculate α as follows:

5 $$\begin{aligned} \mu _1(F_L\cdot \cos \alpha + F_N \cdot \sin \alpha ) = F_N\cdot \cos \alpha - F_L\cdot \sin \alpha \end{aligned}$$

**Fig. 5:**
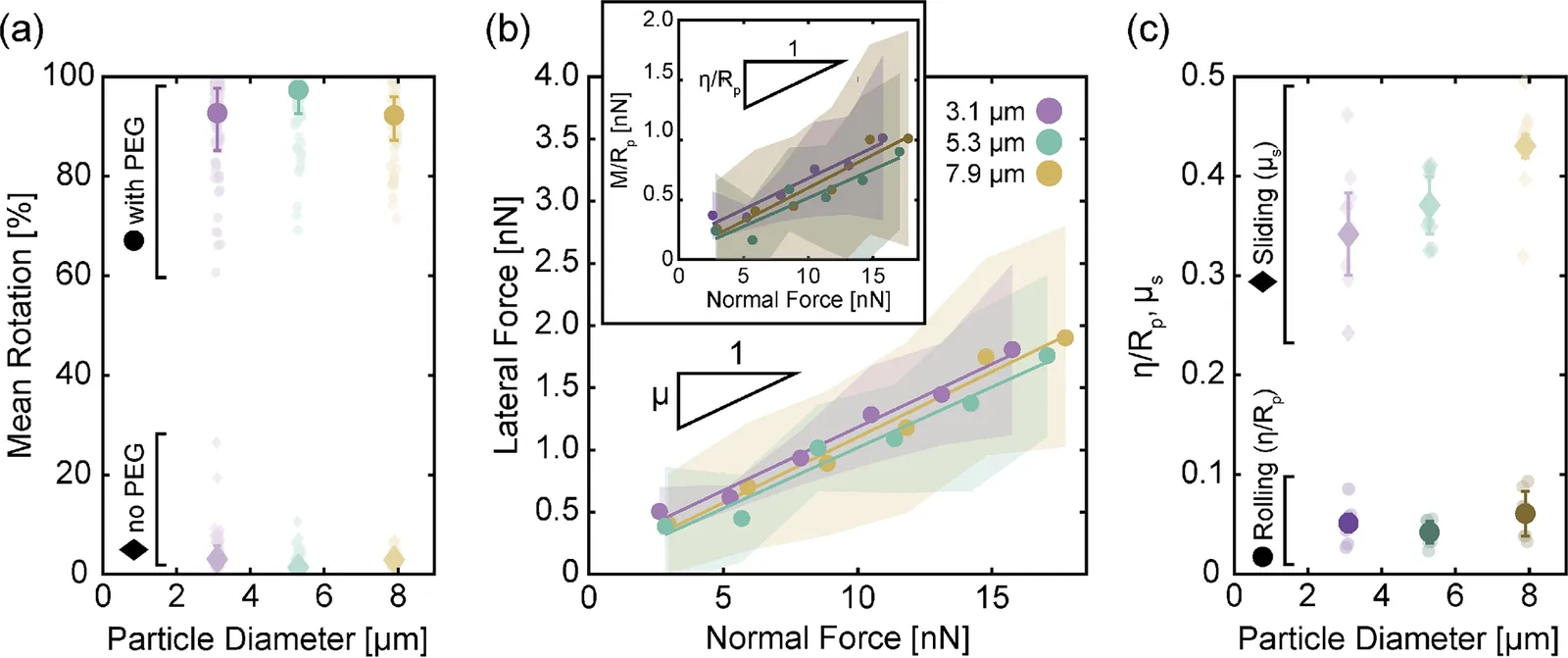
Rotation and friction of free PMMA particles of different sizes against rough substrates before and after surface functionalization of the holder. a) Rotation of free particles (3.1, 5.3, and 7.9 μm) using a holder without any surface modification and with PEG functionalization, expressed as a percentage of total angular displacement during a friction loop compared to that of pure rolling without slip. b) Lateral force versus normal force for free particles (3.1, 5.3, and 7.9 μm) using a holder after PEG functionalization (μ), as well as the calculated lateral force $M/R_p$ that the particle experiences from the substrate without the contribution of the particle–holder contact ($\eta /R_p$). c) Friction coefficients, extracted from the linear fit of the lateral versus normal force plots in b)

Finally, we can use Eq. 3 to calculate $M/R_p$ for experiments with >50 % mean rotation and extract $\eta /R_p$ from the linear fit of $M/R_p$ versus $F_N$, as shown in Fig. 5b. Note that this model assumes simplified, point-like contacts under a rigid-body approximation. We thus expect it to be applicable to the stiff PMMA-silica or PMMA-resin contacts used in this work, while its extension to softer substrates will require modifications.

## Results and Discussion

### Probe Fabrication

We assemble the free-colloidal probes from commercially available tip-less AFM cantilevers and custom-fabricated holders using a workflow similar to how standard fixed-colloidal probes are prepared. Users familiar with colloidal probe LFM can therefore readily implement this method without a steep learning curve.

The holder geometry is defined by a 3D CAD model file, in which only the cavity width and depth are scaled according to particle diameter. Using this approach, we reliably trap particles with diameters of 3.1, 5.3, and 7.9 μm, and have previously demonstrated measurements with 12 μm particles [38].

To improve robustness and throughput, we print holders in their final assembly orientation, using sacrificial support cones and a wide base (Fig. 1a,b). This geometry prevents photoresist entrapment within the cavity and simplifies post-processing. Multiple holders (of one or multiple geometries) can be printed on a single substrate and stored for extended periods. The workflow also allows holders to be shipped, if access to a 2PP-DLW system is not available. During assembly, the supports detach cleanly, yielding well-aligned holders with reproducible geometry (Fig. 1c-e).

These design and fabrication modifications significantly reduce manual handling, improve yield, and make the method suitable for routine use [37].

### Controlling Particle–Holder Friction via PEG Functionalization

A defining feature of the method is the possibility to apply a controlled normal force onto the particle. This implies that during rolling, the particle experiences friction ($\mu _1$, see Fig. 4) at the contact with the holder. The particle–holder interactions determine whether the particle rolls or slides, and contributes to the total force that is measured. In the original design [38], $\mu _1$ was not independently controllable and could dominate the torque balance, limiting the measurement of contacts with low particle–substrate rolling friction.

To decouple internal dissipation from the particle–substrate interaction of interest, we selectively modify the holder surface. We deposit a thin gold layer and functionalize it with PEG-thiol, forming a dense polymer brush within the holder cavity. This modification is implemented after probe fabrication and does not alter the particle or substrate chemistry. Independent friction measurements on flat reference surfaces confirm a substantial reduction in sliding friction upon PEG functionalization, with longer PEG chains providing the strongest effect (Fig. 2 and Fig. S3). Figure 2b shows example lateral versus normal force plots for the pristine IP-S photoresin surface and the PEG10K-coated surface, from which μ was extracted. The friction coefficient μ that we determine here is equivalent of the particle–holder friction coefficient $\mu _1$, which we use later to calculate the rolling friction coefficient $\eta /R_p$. Importantly, all measurements use an unmodified PMMA particle. The PEG layer is thus a very efficient lubricant also under asymmetric conditions [51, 52]. The narrow distribution of friction coefficients indicates a homogeneous coating. Ellipsometry confirms a brush thickness of approximately 8 nm for the PEG10K, consistent with reported values [53].

By reducing $\mu _1$, we suppress internal dissipation within the holder, enabling rolling motion on substrates with low intrinsic friction. This surface modification is therefore recommended as a standard step when performing rolling friction measurements with free-colloidal probes.

### Polymeric Microparticles with Tunable Size and Anisotropic Fluorescence

While the free-colloidal probe method can be applied to any spherical microparticle, the combination of AFM with inverted fluorescence microscopy enables direct correlation between particle rotation and friction forces. This requires particles with anisotropic fluorescence for rotational tracking. Several strategies to generate fluorescent anisotropy have been reported, including photo-bleaching [30], off-center fluorescent cores [54], and surface functionalization [49, 55–58]. Many of these approaches hinder the independent control over surface chemistry and friction. Previously, we have demonstrated that the rolling friction method can be employed on smooth and rough silica particles, with and without additional polymer coatings [38] via post-modification of the surface. Here, we introduce a synthesis route that intrinsically produces fluorescence anisotropy while preserving a smooth, spherical geometry.

Using microfluidic-droplet generation followed by solvent evaporation (Fig. 3a), we obtain smooth polymer particles with diameters between 3 and 8 μm and narrow size distributions (Fig. 3b). During solidification, quantum dots assemble at the oil–water interface, creating inhomogeneous surface fluorescence suitable for rotational tracking (Fig. 3c). SEM and AFM characterization confirm spherical shape and an almost perfectly smooth surface (Fig. 3d–f).

This synthesis route expands the range of particle sizes and materials compatible with the method (beyond the reported silica particles [38, 49]) while preserving native surface properties. We can conveniently tune the particle size via polymer concentration and flow-rate ratios, enabling systematic studies across particle sizes.

### Friction Measurement of PMMA Particles of Different Sizes

We demonstrate the method by measuring friction and rotation of 3.1, 5.3, and 7.9 μm PMMA particles before and after the surface modification of the holders.

With unmodified holders, the rotation of particles remains below <5% for all sizes (Fig. 5a), indicating that the friction between particles and holders $\mu _1$ dominates the torque balance. We then repeated the friction measurements after PEG functionalization of the holders. The PEG layer significantly reduces particle–holder friction, leading to a transition to rolling-dominated motion with mean rotations exceeding 90 % (Fig. 5a). The mean rotation is consistent across the investigated normal force range (2–18 nN), as shown in Fig. S4.

The friction coefficient μ is measured at the same time (Fig. 5b and Fig. 5), representing the combined resistance of particle–substrate and particle–holder interactions. Using the independently quantified $\mu _1$ value from Fig. 2a, and the analytical model described earlier 2.10, we extract the rolling resistance of the particle $M/R_p$, as shown in the inset in Fig. 5b. From the slope of the linear fit of the $M/R_p$ versus normal force data, we can determine the true particle–substrate rolling friction $\eta /R_p \approx 0.05$ for all three particle sizes (Fig. 5b,c). Representative videos are provided in the Supplementary Information(Videos S2–S7).

For comparison, unmodified holders exhibit sliding-dominated motion and their friction coefficients are significantly higher (Fig. 5c and Fig. S5). Since the extracted rotation of these particles is very low, we denote these coefficients as sliding friction $\mu _s$, providing direct comparison of the difference in resistance of the same types of particles, depending on their mode of motion. In this specific case, $\mu _{s}$ ≈ 0.35–0.45, which is comparable to the sliding friction coefficient of the PMMA particle against the pristine photoresin surfaces (see Fig. 2a) of $\mu _{s} \approx$ 0.36. Consequently, for non-functionalized holders, the motion of the particles is governed by sliding, preventing access to information on rolling friction.

Together, these results establish and demonstrate a clear workflow for rolling friction measurements using fCP-LFM, in particular showing how to fabricate and scale the holder geometry, prepare particles with suitable fluorescence anisotropy, suppress internal dissipation via holder functionalization, and extract rolling friction under controlled load.

Across the three particle sizes studied, the extracted rolling friction coefficients $\eta /R_p$ fall within a narrow range. This is expected as the definition of the rolling friction coefficient explicitly accounts for particle size—an observation that dates back to Coulomb [59]—and is consistent with the results presented here (Fig. 5c).

In contrast, the reported sliding friction coefficients $\mu _s$ moderately increase with particle size. Since the materials and surfaces of the tribopair remain unchanged (except for the particle size), one would generally not anticipate such a variation. However, in the present case, changing the radius of the particles might modify how the two surfaces interact.

In particular, when comparing the particle size with the roughness of the substrate (see Fig. S2), the lateral spacing of the asperities is comparable to the particle radii. This suggests that particle–substrate interactions may change with particle size, providing plausible mechanisms for the observed variation in $\mu _s$. Related effects have previously been reported, for example, by varying the asperity density of a substrate at a fixed particle size, which altered the contact mechanics and adhesion [60, 61]. These considerations highlight an intriguing aspect of the system that could be explored in further detail, especially when also considering rolling.

The present data primarily serve to demonstrate the capability of the method, rather than to establish definitive scaling laws. The systematic exploration of the effects of size, roughness, adhesion, and material represents a natural direction for future studies using this platform.

## Conclusion

Starting from our previous implementation [37, 38], we are developing the fCP-LFM method into a versatile platform for quantitative measurements of rolling friction at the microscale. We explore a particle-fabrication technique that broadens the library of materials and sizes available to be investigated, while still maintaining the anisotropic fluorescence essential for rotational tracking. The architecture of the holder design is reconsidered to accommodate particles of different sizes and improve the assembly workflow through simple changes in the CAD model. Finally, we identify the internal friction between the particle and the holder as the key factor that can suppress rolling-dominated motion in certain cases. We develop a strategy to lower the friction at the particle–holder contact in order to improve both the sensitivity of our system and the access to previously unattainable regimes. All these changes combined allow us to overcome several key limitations of earlier implementations. It is, however, worth mentioning that the extraction of the rolling friction coefficients presented here relies on the simplified mechanical model of Eqs. 3–5 which imposes a torque balance assuming two singular contact points between the probe particle and the holder and the particle and the substrate, respectively. In reality, both these contacts will have a finite contact area, whose geometry, evolution, and stress distribution within, will affect dissipation. Focusing on the rolling particle–substrate contact patch, rolling friction can have different physical origins, deriving from hysteresis between the advancing and trailing regions of the contact. This hysteresis can for instance originate from the viscoelastic response of the materials upon contact and deformation, or from asymmetry in adhesion, when present [62–65]. We thus envisage future studies precisely aimed at using our technique to disentangle different physical origins of rolling friction. We propose extending our measurements to softer viscoelastic substrates to increase both contact area and dissipation, as well as expanding previous studies on adhesive particles [38]. Moreover, our workflow could greatly benefit from computational models that describe contacts in more realistic ways, to account for finite contact areas and their dependence on surface topography.

These advances in particular allow us to access rolling-dominated motion and extract true particle–substrate rolling friction for particles between 3 and 8 μm in diameter, demonstrating the method’s applicability beyond the previously studied model system. Using PEG-functionalized holders, free particles exhibit >90 % rolling displacement for all tested particle sizes, whereas unmodified holders yield <5 % rolling. The measured rolling friction coefficients do not vary with the radius of the particles, in contrast to the weak but measurable size dependence observed for sliding contacts. We can attribute the measured variation of the sliding friction coefficient with probe-particle size to the limited multi-asperity nature of the contact between the PMMA particles and the rough substrate. In the applied normal load range, the Hertzian contact radii of the probe particles are on the same length scale of the dimensions of the nanoparticle asperities on the substrate (both on the order of 10 nanometers, see Figure S6). Because of these dimensions, the probe particle is only in contact with a small number of asperities. Therefore, by increasing the probe-particle size the real contact area between the particle and the substrate increases, i.e., the effective number of contacting asperities grows. Although the contact between the probe and the substrate is in principle a multi-asperity contact, the very small number of asperities is insufficient to result in macroscopic contact statistics, and thus the true contact area depends on the probe-particle radius. Therefore, as expected for multi-asperity contacts, the friction coefficient depends on the real contact area, and we observe an increase upon using larger probe particles that can effectively interact with a greater number of asperities. Conversely, the rolling friction coefficient is already normalized by the particle radius, leading to a value that is essentially independent of probe-particle size. The scaling of the sliding and rolling friction coefficients with real contact area is therefore an extremely interesting direction for future research, where systematic variations in a limited multi-asperity contact scenario may elucidate distinct fundamental physical mechanisms of these two dissipation modes.

Beyond improving measurement accuracy and throughput, the presented workflow broadens the scope of systems for which rolling friction can be quantified. The approach is compatible with a wide range of particle materials, sizes, and surface chemistries, and could be extended to structured or viscoelastic substrates. We anticipate that these capabilities will support systematic studies of surface roughness, adhesion, lubrication layers, and particle–particle contacts in regimes where rolling plays a dominant role. Ultimately, this platform provides a foundation for linking nanoscale rolling phenomena to macroscale suspension behavior and for improving predictive models for dense, frictional materials. We expect that this platform will facilitate routine rolling friction measurements in laboratories already equipped with colloidal probe LFM, lowering the barrier to systematic studies of rolling-dominated contacts.

## Supplementary Information

Below is the link to the electronic supplementary material.Supplementary file 1 (pdf 1625 KB)Supplementary file 2 (mp4 22536 KB)Supplementary file 3 (avi 3520 KB)Supplementary file 4 (avi 1706 KB)Supplementary file 5 (avi 1140 KB)Supplementary file 6 (avi 2223 KB)Supplementary file 7 (avi 1646 KB)Supplementary file 8 (avi 4129 KB)

## Data Availability

Data are available from the corresponding authors upon reasonable request. The STL files for the printing of the holder are publicly available at https://doi.org/10.3929/ethz-c-000792163
