# Supplementary material for: Free-Colloidal Probe Lateral Force Microscopy (fCP-LFM) for Nanotribology of Sliding and Rolling Contacts
Source: Tribol Lett. 2026 May 19;74(3):58. doi: 10.1007/s11249-026-02147-8 (PMC13186866; doi:10.1007/s11249-026-02147-8)
Supplement: Supplementary file 1 — (pdf 1625 KB) [file 11249_2026_2147_MOESM1_ESM.pdf]

Free-Colloidal Probe Lateral Force Microscopy (fCP-LFM) for Nanotribology of Sliding and Rolling Contacts

Simon Scherrer[1], Noé Margni[1], Kristian Skailand[1], Minghan Hu[2], Robert W. Style[1], Shivaprakash N. Ramakrishna[1*], Lucio Isa[1*]

[1]Department of Materials, ETH Zürich, Leopold-Ruzicka-Weg 4, Zürich, 8093, Zürich, Switzerland.
[2]Department of Mechanical and Process Engineering, ETH Zürich, Leopold-Ruzicka-Weg 4, Zürich, 8093, Zürich, Switzerland.

*Corresponding author(s). E-mail(s): shivaprakash.ramakrishna@mat.ethz.ch; lucio.isa@mat.ethz.ch;
Contributing authors: simon.scherrer@mat.ethz.ch; nmargni@ethz.ch; kristian.surdal.skailand@gmail.com; minghu@ethz.ch; robert.style@mat.ethz.ch;

1 Supporting Figures

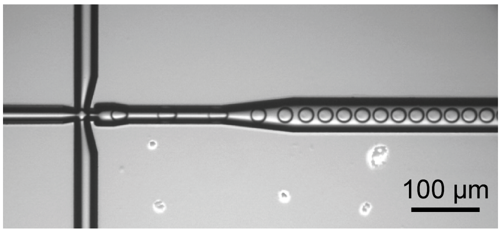

Fig. S1 Optical microscopy image of the flow-focusing junction of the microfluidic chip that produces the monodispersed oil droplets.

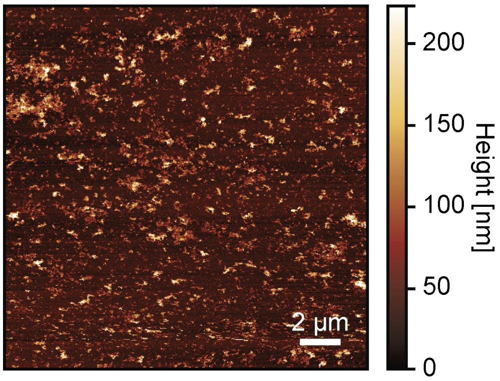

Fig. S2 Tapping mode AFM scan of a rough substrate, prepared via electrostatic adsorption of 8 nm silica nanoparticles onto a glass slide.

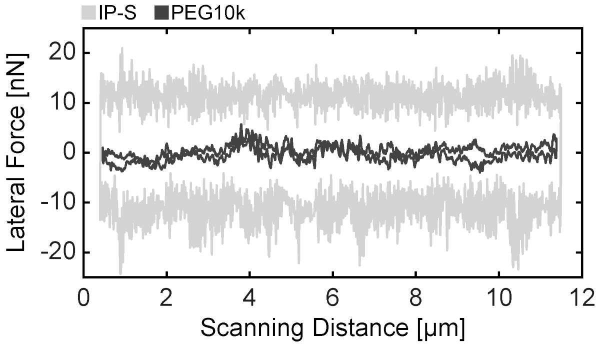

Fig. S3 Example sliding friction loops of a PMMA particle on bare IP-S and PEG10K functionalized IP-S, at ∼35 nN normal force.

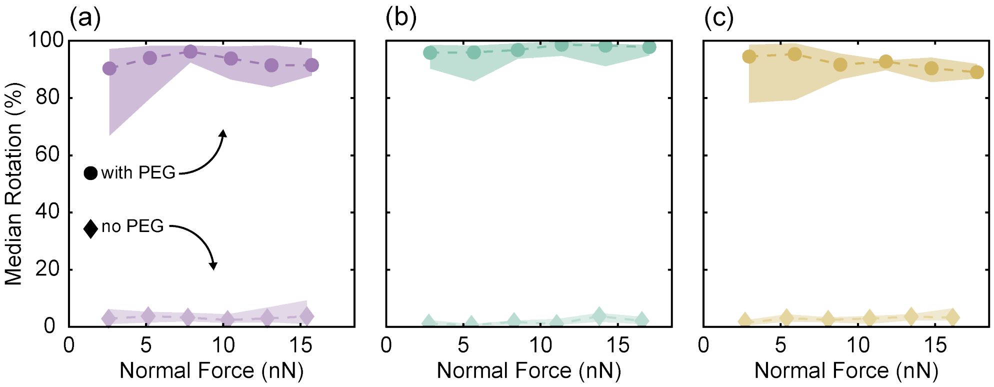

Fig. S4 Mean Rotation of different particles as a function on applied normal force before and after functionalizing the probe with PEG-brushes. a) 3.1 μm b) 5.3 μm c) 7.9 μm.

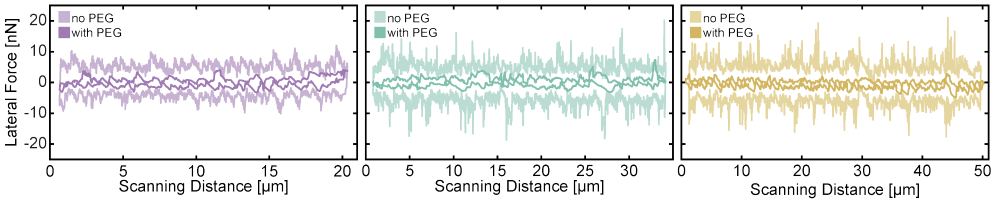

Fig. S5 Example friction loops of different particles at ∼17 nN normal force before and after functionalizing the probe with PEG-brushes. a) 3.1 μm b) 5.3 μm c) 7.9 μm.

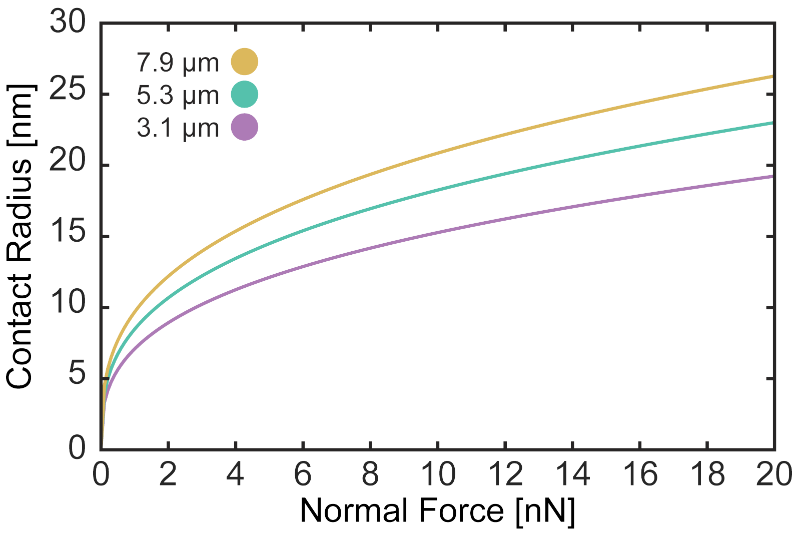

Fig. S6 Calculated Hertzian contact radius for the different probe particles at the PMMA silica contact.

2 Supporting Movies

- **Movie S1: Representative confocal microscopy movie illustrating the formation of a polymeric microparticle.** Monodisperse droplets containing chloroform, PMMA and fluorescent quantum dots are generated in a flow-focusing device and solidify as chloroform diffuses into the surrounding aqueous SDS solution, leading to polymer precipitation. The movie shows the temporal evolution of a droplet (10 mg/mL PMMA, water flow rate: 4 μL/min, oil flow rate: 1 μL/min) during solvent removal and particle formation. Imaging was performed using a confocal microscope (Yokogawa CSU-W1, Japan; Axio Observer D1, 63 $\times$ NA = 0.75 objective, Zeiss, Germany; Prime 95B, Teledyne Photometrics, USA; laser: 488 nm, filter: 510-540 nm, exposure time: 100 ms).
- **Movies S2–S7: Representative confocal microscopy movies of fCP-AFM friction experiments performed on individual PMMA particles of different diameters, before and after surface functionalization.** In all experiments, a single friction loop was performed by laterally translating the particle at a constant velocity over the indicated scan distance. Imaging was performed using a confocal microscope (Yokogawa CSU-W1, Japan; Axio Observer D1, 63 $\times$ NA = 0.75 objective, Zeiss, Germany; Prime 95B, Teledyne Photometrics, USA; laser: 488 nm, filter: 510-540 nm, exposure time: 50 ms).
  - **Movie S2:** fCP-AFM experiment with a 3.1 μm particle before surface functionalization. Translation velocity: 2.6 μm/s; scan distance: 19.5 μm.
  - **Movie S3:** fCP-AFM experiment with a 5.3 μm particle before surface functionalization. Translation velocity: 4.4 μm/s; scan distance: 33.3 μm.
  - **Movie S4:** fCP-AFM experiment with a 7.9 μm particle before surface functionalization. Translation velocity: 6.6 μm/s; scan distance: 49.6 μm.
  - **Movie S5:** fCP-AFM experiment with a 3.1 μm particle after surface functionalization. Translation velocity: 2.6 μm/s; scan distance: 19.5 μm.
  - **Movie S6:** fCP-AFM experiment with a 5.3 μm particle after surface functionalization. Translation velocity: 4.4 μm/s; scan distance: 33.3 μm.
  - **Movie S7:** fCP-AFM experiment with a 7.9 μm particle after surface functionalization. Translation velocity: 6.6 μm/s; scan distance: 49.6 μm.
